# Functional connectivity between prefrontal and parietal cortex drives visuo-spatial attention shifts

**DOI:** 10.1016/j.neuropsychologia.2017.02.024

**Published:** 2017-05

**Authors:** Klaartje Heinen, Eva Feredoes, Christian C. Ruff, Jon Driver

**Affiliations:** a Institute of Cognitive Neuroscience, University College London, London WC1N 3AR, UK; b Wellcome Trust Centre for Neuroimaging, University College London, London WC1N 3BG, UK

**Keywords:** Attention shifts, Frontal eye-fields, Thetaburst TMS, fMRI

## Abstract

It is well established that the frontal eye-fields (FEF) in the dorsal attention network (DAN) guide top-down selective attention. In addition, converging evidence implies a causal role for the FEF in attention shifting, which is also known to recruit the ventral attention network (VAN) and fronto-striatal regions. To investigate the causal influence of the FEF as (part of) a central hub between these networks, we applied thetaburst transcranial magnetic stimulation (TBS) off-line, combined with functional magnetic resonance (fMRI) during a cued visuo-spatial attention shifting paradigm.

We found that TBS over the right FEF impaired performance on a visual discrimination task in both hemifields following attention shifts, while only left hemifield performance was affected when participants were cued to maintain the focus of attention. These effects recovered ca. 20 min post stimulation. Furthermore, particularly following attention shifts, TBS suppressed the neural signal in bilateral FEF, right inferior and superior parietal lobule (IPL/SPL) and bilateral supramarginal gyri (SMG). Immediately post stimulation, functional connectivity was impaired between right FEF and right SMG as well as right putamen. Importantly, the extent of decreased connectivity between right FEF and right SMG correlated with behavioural impairment following attention shifts.

The main finding of this study demonstrates that influences from right FEF on SMG in the ventral attention network causally underly attention shifts, presumably by enabling disengagement from the current focus of attention.

## Highlights

• Thetaburst stimulation to the right FEF temporarily impairs bilateral attention shifts.
• Lateralised behavioural deficits in the contralateral hemifield are observed when cued to maintain attention.
• These effects recover ca. 20 min post stimulation.
• During shifts, neural activity is suppressed following right FEF TBS in the dorsal attention network and supramarginal gyri.
• Influences from right FEF to SMG causally underlie attention shifts, presumably by enabling disengagement from current focus.

## 1 Introduction

Successful interaction with the environment requires a balance between holding attention when stimuli are within-, and shifting attention when behaviourally relevant stimuli appear outside the current focus of attention. These processes rely on distributed fronto-parietal networks and a distinction is often made between a bilateral dorsal attention network (DAN) and a more ventral, mainly right lateralised, attention network (VAN). Of these networks, the DAN is thought to control selective attention based on endogenous goals, whereas the VAN would be specifically recruited when behaviourally relevant stimuli appear in a non-selected location, prompting a shift in attentional focus (Corbetta and Shulman, 2002, Corbetta and Shulman, 2011, Fox et al., 2006, Gitelman et al., 1999, Yantis et al., 2002). Recent studies however, suggest this is not a strict dichotomy and both networks may process both exogenous and endogenous signals (Macaluso and Doricchi, 2013). In addition to these fronto-parietal networks, a fronto-striatal system is also implied in attention shifting, particularly when attention shifts are unpredictable (Shulman et al., 2007).

Within the DAN, the frontal eye-fields (FEF) form a key structure. Several studies, which combined stimulation methods and imaging, have established that the FEF plays a causal role in the mechanism underlying endogenously guided selection of locations or features in a visual scene by sending biasing signals to the visual cortex (Blankenburg et al., 2010, Ekstrom et al., 2009, Heinen et al., 2013, Moore et al., 2003, Morishima et al., 2009, Ruff et al., 2006, Taylor et al., 2007). Other, TMS-only studies have also shown that the FEF controls (visuospatial) attention shifts (Duecker et al., 2013, Grosbras and Paus, 2002, Grosbras and Paus, 2003). However, these TMS studies were not combined with imaging techniques and how FEF may influence remote brain regions during attention shifts is therefore unknown.

The process of attention shifting involves disengagement from the current focus of attention and orienting towards the newly selected location (Corbetta et al., 2000, Posner et al., 1980). One or both of these processes may require the integration of signals between regions in the DAN and the VAN (Macaluso et al., 2002, Vossel et al., 2012) and/or the striatum (Cools et al., 2004). Resting state studies suggest that the Inferior Frontal Junction (IFJ) could play a general role in coordinating the recruitment of these networks (He et al., 2007) but underlying mechanisms are unknown. Considering the causal role of FEF in visuospatial attention shifts, we ask the specific question whether the FEF influences integration of signals within and/or between attention networks and visual cortex in the context of visuospatial attention shifting.

**Fig. 1:**
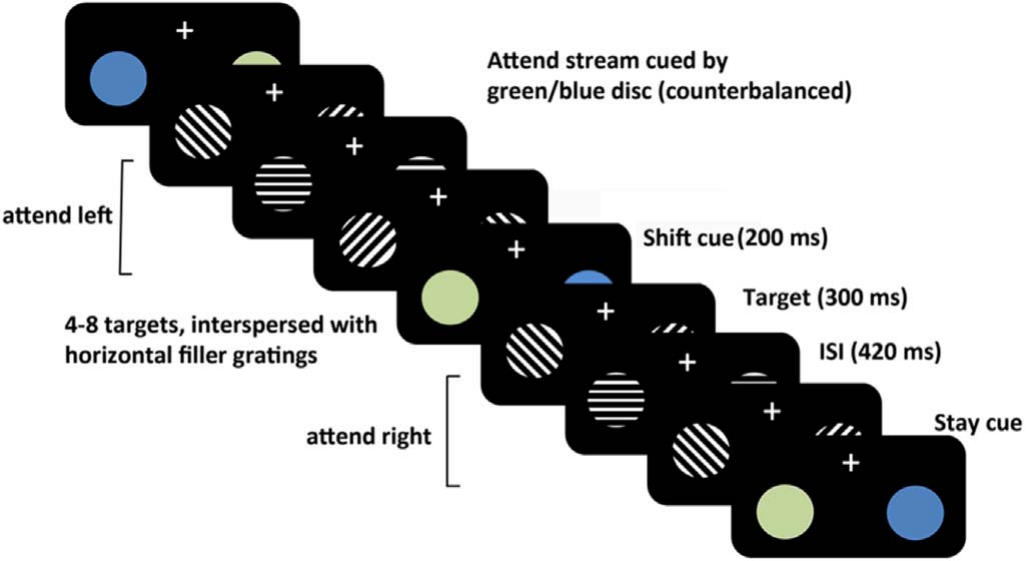
Experimental procedure: During the visuospatial attention shifting task, participants had to make perceptual judgements on clockwise or anti-clockwise tilted gratings presented in bilateral streams, while holding their gaze on a central fixation cross. Colour cues embedded in the stream instructed participants to maintain their focus on the currently attended stream or shift to the stream in the opposite hemifield. The ‘shift’ or ‘stay’ cues appeared after a time interval of variable duration with 50% probability. On different days and counterbalanced across participants, either real or Sham TBS would be administered over the right FEF in between sessions.

We investigate this hypothesis in the present study, by temporary perturbation of neural activity in the right FEF using off-line thetaburst TMS (TBS) during a visuo-spatial attention shifting paradigm (see Fig. 1) combined with fMRI recordings. Based on earlier TMS studies we expected interference of processing in the right FEF to affect the ability to appropriately shift attention. By measuring the effects on BOLD before and after stimulation, we aim to elucidate causal influences of the right FEF on other regions within the attention networks and/or visual cortex during conditions when attention was maintained or shifted to the other hemifield.

## 2 Materials and methods

### 2.1 Participants

16 healthy participants (6 female, age range 19–34), all right handed and with normal or corrected vision participated in the study. Following screening for medical contra-indications to MRI and TMS all provided informed consent in line with the guidelines set by the local ethics committee (National Hospital for Neurology and Neurosurgery & Institute of Neurology Joint Research Ethics Committee, London, UK). Two participants were excluded from analysis due to technical problems during the experiment, leaving 14 participants for analysis.

### 2.2 Stimuli and procedure

Participants undertook a spatial attention shifting task in the scanner while accuracy of perceptual discrimination of grating orientation (clockwise/anti clockwise) (see Fig. 1) and reaction times were recorded. Per block, four equiprobable mini-block conditions were generated through the factorial combination of cueing type preceding the mini-block (‘stay’ or ‘shift’) and attended hemifield (left or right). Separate scanning experiments were conducted with off-line TMS applied outside the scanner in between four task blocks, either as effective thetaburst TMS (TBS) over the right FEF or non-effective Sham stimulation with the same frequency.

The stimulus stream comprised oriented gratings which were tilted along the vertical plane. Prior to onset of each experiment, but already in the scanner, the degree in tilt of the target gratings were adjusted (varying between 1 and 4°) with a staircase procedure to ensure that proportion correct scores were ca. .8. Participants were instructed to attend (covertly) either the left- or right hemifield stream of stimuli depending on a cue (200 ms) consisting of two coloured discs (green and blue) presented bilaterally on the location and with the dimensions of the ensuing stimuli. Participants were spatially cued either by the green or blue disc (instructions counterbalanced across participants). The appearance of a subsequent cue combination would end the continuous stimulus steam (mini-block), which instructed participants, with equal probability, to maintain their focus on the currently attended hemifield (stay) or shift attention to the stream in the opposite hemifield (shift). The probability of a cue combination instructing either to maintain or shift attention was 50%. A stimuli stream between cues consisted of minimal 4 and maximal 8 target gratings, interspersed with 1–3 non-target horizontal gratings to ensure a continuous stream of stimuli but to avoid predictability of target- and cue events. Participants were instructed to indicate the tilt of the grating of the target stimuli (clockwise or anti clockwise to the vertical plane) on an MR compatible response box with a right-hand key press, using the index- or middle finger with stimulus-contingencies counterbalanced across blocks. Gratings were 3° of visual angle in diameter and appeared in the lower visual field 6° from the display midline at an eccentricity of 10° from the centre. The inter-stimulus interval was 300 ms and stimulus duration 420 ms. Throughout the experiment, participants were instructed to retain fixation on a constantly present central fixation cross. In order to avoid anticipation, the cue- (first)target interval was jittered between 200–1500 ms and between-target interval varied between 1500–3000 ms. This would yield mini-blocks of either left- or right attended stimulus streams, uninterrupted by cues, of on average 15.5 s. A block would contain 28 mini-blocks with four one-minute breaks randomly interspersed throughout the experimental block, during which only a fixation cross was displayed. These one-minute breaks were used as an internal baseline during the fMRI analysis (see below). In total a block would last ca. 14 min. An experiment consisted of two blocks pre-TBS/Sham and two blocks post. Data was analysed from the block immediately preceding stimulation (block pre TBS; the first block was excluded from the analysis testing for TBS effects but used to test main task effects and to define ROIs) and from the two blocks following stimulation (block post -TBS1 and post TBS2).

### 2.3 Localisation of TMS sites

Scalp coordinates for the stimulation sites were first located outside the scanner via the Brainsight Frameless stereotaxic system and software package (Rogue Research, Montreal, Canada), using the native space of each participant's own T1-weighted anatomical MR. image. The FEF was identified in the right hemisphere for each participant (and then marked on their scalp) based on the following anatomical landmarks: in the posterior middle frontal gyrus, immediately ventral to the junction of superior frontal sulcus and ascending limb of precentral sulcus, as described in earlier work from our laboratory (Ruff et al., 2006). The selected FEF site, after normalizing to MNI (Montreal Neurological Institute) space, corresponded to mean XYZ coordinates of 27, 3, 57 (±1, 1, .5 SEM). See Supplementary Fig. S1 for sites marked on each individual's native space anatomical scan.

### 2.4 TMS

Preceding the fMRI experiment and on a separate day, individual active motor thresholds (aMT) were obtained via stimulation over the right M1 ‘hotspot’, while participants were asked to contract the index-finger and thumb. aMT's were set to an intensity that elicited 5 out of 10 MEPs measured from the first dorsal interosseus with an amplitude of at least 50 μV. TBS stimulation intensity would subsequently be set to 80% of aMT, unless this exceeded 50% of maximal stimulator output, in which case TBS stimulation would be capped at 50% of maximal stimulator output.

During thetaburst (TBS) stimulation, the TMS coil was positioned over the marked location with the handle oriented 45° from the vertical midline in a posterio-medial direction. A Super Rapid stimulator (Magstim, Whitland, UK) was used to generate TMS, with a figure-of-eight coil with a wing diameter of 70 mm. The TBS protocol consisted of a continuous application of short burst of 3 pulses at 50 Hz delivered at 5 Hz for 40 s, yielding 600 pulses in total. This continuous TBS has been shown to induce inhibitory effects on cortical excitability, of which the mechanisms are suggested to rely on long-term depression-like plastic changes in cortical synapses (Huang et al., 2005) and thus produce effects over a prolonged time period. During Sham stimulation one wing of the figure-8 TMS coil was held on the same marked location on the skull, but now in a perpendicular fashion, preventing any current to flow into the brain, but eliciting the same acoustic sensation. The slight difference in tactile sensation between real- and Sham stimulation was deemed negligible especially as the TMS was delivered off-line.

The time between stimulation and resuming of scanning would vary between 5–10 min. The order of the TBS and Sham conditions was counterbalanced across subjects, i.e., each subject had a different sequence of conditions with real and Sham stimulation. The two stimulation conditions were separated by at least 5 days.

### 2.5 Functional magnetic resonance imaging

Whole-brain T1-weighted anatomical images were acquired in a separate session before the functional MRI experiment using an eight-channel phase array coil and a 3D modified driven equilibrium fourier transform (MDEFT) sequence with an isotropic resolution of 1 mm3 (Deichmann et al., 2004).

During scanning, the visual display was back-projected onto a screen spanning approximately 30°×22° of visual angle, viewed via a mirror mounted on top of the MR head coil. Functional images were acquired on a 3 T MR system (Siemens Allegra, Erlangen, Germany), with a single channel receive head array. T2*- weighted echo planar image (EPI) volumes were acquired every 1.98 s covering the whole brain down to the cerebellum (33 transversal slices; α=−10°; repetition time (TR), 60 ms; echo time (TE), 30 ms; 64 matrix; voxel size 3.00×3.00×3.75 mm; 2.5 mm slice thickness plus a slice distance of 50%). Each fMRI block included 450 volumes.

Throughout scanning, eye position, blinks and pupil diameter were monitored with an ASL 504 Remote Infrared Eyetracker (Applied Science Laboratories, Bedford, USA) at 60 Hz via long-range optics. Deviations from fixation larger than 1° were considered as saccades. Visual inspection confirmed participants adhered well to central fixation. Analysis of the collected eye data showed that saccades occurred <4% of the trials. A paired *t*-test comparing number of saccades before and after right FEF TBS or between right FEF TBS and Sham revealed no significant difference (p>.6) and no trials were excluded. Blinks were included in the fMRI analysis as regressors of no interest (see below). Participants wore earplugs to reduce acoustic noise from the scanner.

All visual stimulation, were controlled using the Cogent toolbox http://www.vislab.ucl.ac.uk/cogent_2000.php) within Matlab (Mathworks, MA, USA).

### 2.6 Data analysis

#### 2.6.1 Behaviour

Accuracy scores (proportion correct) and reaction times (RT) were calculated from the recorded responses and to account for speed-accuracy trade-offs, inverse efficiency scores were derived from these (RT divided by proportion correct).

#### 2.6.2 Functional MRI data

The analysis of imaging data was undertaken using SPM8 (http:// www.fil.ion.ucl.ac.uk/spm). The first six volumes of each fMRI run were discarded to allow for T1 equilibration effects. All subsequent volumes were realigned to the seventh volume to correct for inter-block movement, and were spatially normalised to MNI standard space. The normalised images were spatially smoothed for the whole-brain analysis (8 mm full width at half-maximum Gaussian kernel) in accord with the standard SPM approach. Low-frequency fluctuations were removed from the analysis using a temporal high-pass filter (128 s cut-off). The pre-processed images were then fed into a General Linear Model. For each block, data were modelled with a separate mini-block regressor for each of the four experimental conditions in the 2 by 2 factorial design (left- or right attention mini-block preceded by ‘stay’ or ‘shift’ cue). The mini-block regressor covered the time including the preceding cue, and the stimulus stream till the appearance of the subsequent cue. Blink onsets were extracted from the eyetracking data, after being identified using a band-pass filter on pupil reflex data (80–1000 ms, 100% signal loss of pupil and corneal reflection). Together with six head movement regressors, the blink onsets were entered as regressors of no interest to account for any blink-related variance in the fMRI data and thereby ensure that this could not contribute to the effects of the experimental conditions on BOLD responses (see also (Heinen et al., 2011)). BOLD responses were modelled by convolution of the regressors with the canonical haemodynamic response function in SPM8 and its temporal derivative.

Masks for small volume correction for most DAN and VAN regions (FEF and SMG) were derived from task activations (contrasting ‘shift’ versus ‘stay’ on data derived from the first block pre-TBS/Sham, which was not included in the main analysis). Anatomical masks were used for IPL, SPL and striatal regions as task activations obtained for these regions pre-stimulation were not sufficiently specific (probability maps taken from a MNI space probabilistic cytoarchitectonic maps derived from human post-mortem studies into the SPM environment (Eickhoff et al., 2005)). For applied masks see Supplementary Fig. S4. After small volume correction, all reported effects passed the threshold of p<.05 family wise error (FWE).

#### 2.6.3 ROI analysis visual cortex

Visual ROIs were defined by clusters that responded positive to the task versus baseline contrast during the first block pre-TBS/Sham (positive task effects; p<.001, uncorrected). The resulting activation maps were then inclusively masked by an MNI space probabilistic cytoarchitectonic maps for MT+ and V1/V2. The location for left and right MT+ and V1/V2 is shown in Fig. 6 in the main text. Parameter estimates for each condition from the main experiment were then extracted for each ROI and averaged across all voxels within the ROIs. This was done for each participant separately and then averaged across participants and condition. ANOVAs including all 4 factors (TMS-type, block, cue-type and hemifield) were subsequently applied to the data for each ROI separately.

#### 2.6.4 Functional connectivity analysis

For each subject individually, coordinates for the right FEF were determined by looking for the closest cluster near the anatomical landmarks as defined above, which responded positively to the task versus baseline contrast. Subsequently, fMRI time series were extracted from a 6 mm sphere centred on these coordinates and were tested per mini-block for coherent fluctuations with all other brain voxels when contrasting the shift versus stay conditions, after main effects for all task conditions were accounted for (PPI; see (Friston et al., 1997)). On the first level, these PPIs were applied to all blocks (pre-stim, post-stim1 and post-stim2 for both the TBS and Sham conditions) and for each participant separately. The first-level images were subsequently taken to the second level and a U-shaped second-level contrast was applied ((pre TBS+post TBS2) – (2×post TBS1)) – ((pre Sham+post Sham2) – (2×post Sham1)) to test for decreases in functional connectivity during the block immediately post TBS compared to Sham.

## 3 Results

Participants undertook a visuospatial attention shifting task in the scanner (see Fig. 1 and Methods for details). In short, participants had to judge the grating tilt (clockwise/counter-clockwise) of target stimuli either on the left- or the right of a bilaterally presented stream according to a combined colour cue, while holding their gaze at a central fixation cross. Following a variable number of targets (maximal 8), a next cue would appear, which indicated either to maintain attention on the currently attended stream (“stay”) or to shift attention to the stream in the other hemifield (“shift”) with 50% probability. After two task blocks in the scanner, off-line TBS or Sham stimulation was applied over the right FEF outside the scanner, followed by two more blocks. This design thus included 4 factors (TMS-type (FEF TBS or Sham), block (pre-TBS/Sham, post-TBS/Sham1 and post-TBS/Sham2), cueing type (stay or shift) and attended hemifield.

### 3.1 Behaviour

**Fig. 2:**
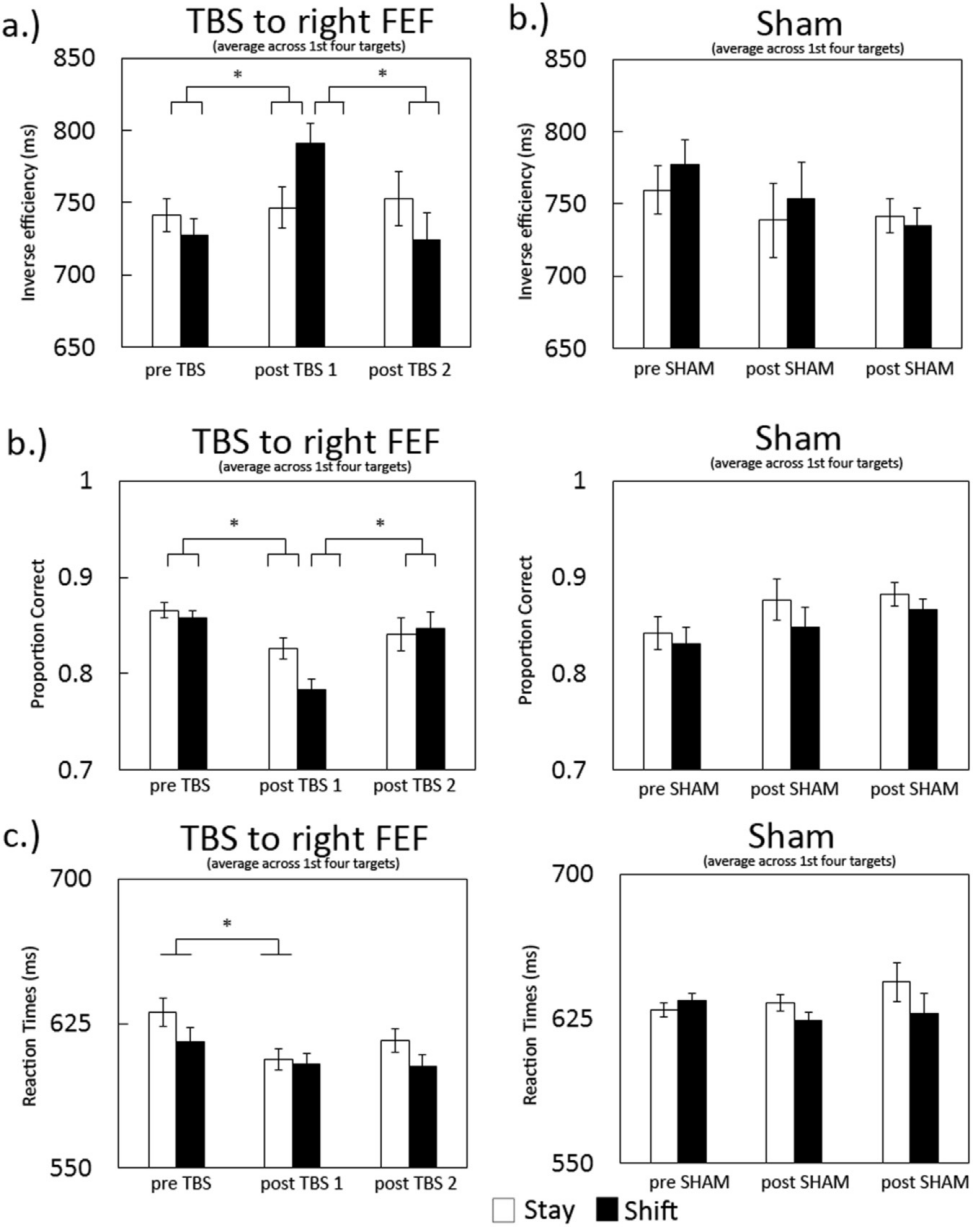
Following right FEF TBS, a general bilateral impairment in task performance was observed for a prolonged period (ie. across several subsequent target stimuli) following a ‘shift’ cue, as reflected in increased inverse efficiency scores (averaged across first four targets post cue (a), which was not observed across targets following a ‘stay’ cue. These effects recovered during the second session ca. 20 min post TBS and were not observed in the Sham condition. The behavioural deficit was mainly due to a decrease in accuracy, predominantly on ‘shift’ trials (b) while reaction times decreased across both conditions (c).

Accuracy (proportion correct) and speed (reaction times, RT) were calculated across the block preceding- and the two blocks following intervention (TBS or Sham to the right FEF). To account for speed-accuracy trade off, inverse efficiency scores (RT divided by proportion correct) were derived from these. A first inspection of the data learned that the effects of TBS lingered longer than just the first target post cue and were prominent for at least four targets post cue (see Supplementary Fig. S2 for effects for the first four targets post cue separately). We therefore averaged efficiency scores across the first four targets post cue as presented in Fig. 2. Scores are displayed for the experimental (right FEF TBS) and control (Sham) data, separated for the trials preceded by a ‘shift’- or a ‘stay’ cue. As no effects of hemifield were observed when performance was averaged across the first four targets, presented data are collapsed across hemifield.

To test for effects of TBS to the right FEF, we applied an ANOVA on the inverse efficiency scores comparing data pre- and immediately post intervention, including the factors TMS-type (TBS or Sham), block (pre TBS/Sham and post TBS/Sham1), cue (stay and shift) and hemifield. This analysis revealed no main effects, but an interaction between TMS-type and block (F(1,13)=6.3; p=.026) and between TMS-type, block and cue (F(1,13)=4.5; p=.05), indicating a differential effect of right FEF TBS compared to Sham stimulation, which depended on the cueing condition. To further investigate this effect, a subsequent ANOVA on the experimental data (FEF TBS) separately, revealed no main effects, but a significant interaction between cue-type and blocks (F(1,13)=8.8 p=.01). This reflected a significant *increase* in inverse efficiency scores, which means an *impairment* in performance specifically for the shift condition (F(1,13)=4.6 p=.05). These interaction effects were no longer present during the second block post stimulation (F(1,13)=.43 p>.5 ns. (see Fig. 2a)).

In order to get a better understanding of the nature of effects on the inverse efficiency scores, performance is presented separately for proportion correct and reaction times in Figs. 2b and 2c. A full ANOVA on the proportion correct scores revealed a significant main effect for cue (F(1,13)=5.9 p=.03) and an interaction between TMS-type and blocks (F(1,13)=11.6 p=.005) and no other significant main effects or interactions. This reflected a general accuracy cost of attention shifting and a differential effect of TBS compared to Sham stimulation. Investigating the effects of TBS, an AVOVA including just the experimental (TBS) data (see Fig. 2b) revealed a main effect of block (F(1,13)=5.6 p=.03) and an interaction between cue and block (F(1,13)=6.5 p=.024). This indicates that TBS to the right FEF decreased accuracy immediately post stimulation and significantly more so following a shift cue (F(1,13)=7.7 p=.016) than during the stay condition. These interaction effects were no longer present during the second block post stimulation (F(1,13)=.77 p>.35 ns).

For reaction times, no main effects were observed except for attended hemifield (F(1,13)=5.9 p=.03), indicating overall faster responses for right hemifield targets. This effect of attended hemifield was not significantly affected by TMS-type (p>.28). A marginal interaction was detected between TMS-type, blocks and cue-type (F(1,13)=3.9 p=.07), indicating a differential effect of TBS depending on cue-type. When we analysed the data for the experimental (FEF TBS) data separately, we found main effects of block (F(1,13)=6.7 p=.02) and hemifield (F(1,13)=6.4 p=.025), but no significant interactions. The main effect of block reflects a significant increase in reaction times following TBS reaching significance for the 'stay' condition (F(1,13)=7.3 p=.018).

In summary, following TBS over the right FEF, we observed an increased behavioural cost of attention shifting reflected in a selective and prolonged (ie. across several targets post cue) impairment of performance on ‘shift’ trials, measured as increased inverse efficiency scores. This effect was mainly driven by a decrease in accuracy immediately following TBS. As reported by other studies, the effects of TBS usually last for ca 20–40 min (Huang et al., 2005). We found that the effects of TBS were no longer significant, when comparing the second block post TBS with the pre TBS block. This indicates that the behavioural effects of TBS effects on attention shifting had recovered during the second block post TBS (ca 20 min post stimulation).

**Fig. 3:**
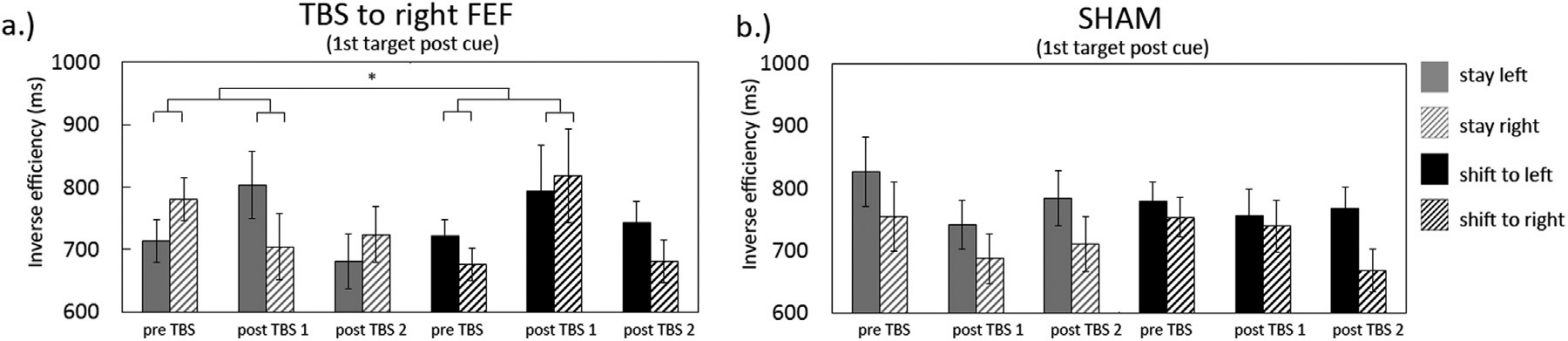
Displayed are right FEF TBS effects on inverse efficiency scores immediately following the cue (including data from the first target post cue only). In contrast to performance averaged across four targets, performance on the first target post cue shows a lateralised impact following a ‘stay’ cue. Performance immediately following a ‘shift’ cue tended to be bilaterally affected (a). These effects were significantly different from the Sham condition (b).

As we were expecting the main impact of TBS on the first target post cue, we ran an additional analysis on the effects of FEF TBS including inverse efficiency scores just on the first targets. An ANOVA including all factors revealed no clear significant main effects. However, significant interactions between TMS-type and blocks (F(1,13)=4.6 p=.05) and between TMS-type, blocks, cue-type and attended hemifield (F(1,13)=4.95 p=.044), indicate a differential effect of TBS, which depended on cue-type and attended hemifield. To further investigate this effect, we subsequently applied an ANOVA including the experimental data only (FEF TBS) as presented in Fig. 3, which shows inverse efficiency scores pre- and post TBS, separated for cue-type and attended hemifield. This analysis yielded no main effects or interaction between block and cue-type. However, an interaction between blocks, cue-type and attended hemifield (F(1,13)=4.8 p=.046) indicates a differential TBS effect, depending both on cue *and* attended hemifield. More specifically, performance on just the first target following a shift-cue displayed a similar pattern of bilateral impairment as observed when performance scores were averaged across the first four targets as described earlier (albeit this trend did not reach significance here (F(1,13)=2.8 p=.12), see Fig. 3b). However, in contrast to the averaged performance, inverse efficiency scores on 'stay' trials were affected in opposite directions between both hemifields, when just the first target post-cue was included in the analysis (block x hemifield, stay condition F(1,13)=6.3 p=.026). This interaction reflected a tendency for impairment on the left- and enhanced performance on the right hemifield, neither of which reached full significance (left T(13)=−1.6 p=.1; right T(13)=1.7 p=.1; see Fig. 3a). The observed effects were no longer detected during the second block post stimulation (F(1,13)=.18 p>.65 ns.). For TBS effects on proportion correct scores and reaction times separately see Supplementary Fig. S3.

In summary, as described above, TBS over the right FEF caused a selective and prolonged bilateral behavioural deficit on successive targets following a 'shift' cue in comparison to targets following a 'stay' cue, as observed when averaging inverse efficiency scores across the first four targets post cue. No significant interactions with attended hemifield were observed. A different pattern was observed when just the first targets immediately post cue were included in the analysis. While performance on post 'shift' targets tended to display a similar bilateral impairment, a lateralised effect emerged for targets immediately following a 'stay' cue, with performance tending to be impaired in the left- and enhanced in the right hemifield. We will further address these findings in the Discussion section of the manuscript.

Because we found prolonged behavioural effects of TBS on attention-shifting, which lingered across multiple targets post cue, the subsequent fMRI analysis was performed by modelling the conditions as miniblocks, including all data between cues, as this analysis yielded more robust effects.

### 3.2 Task effects on BOLD

**Fig. 4:**
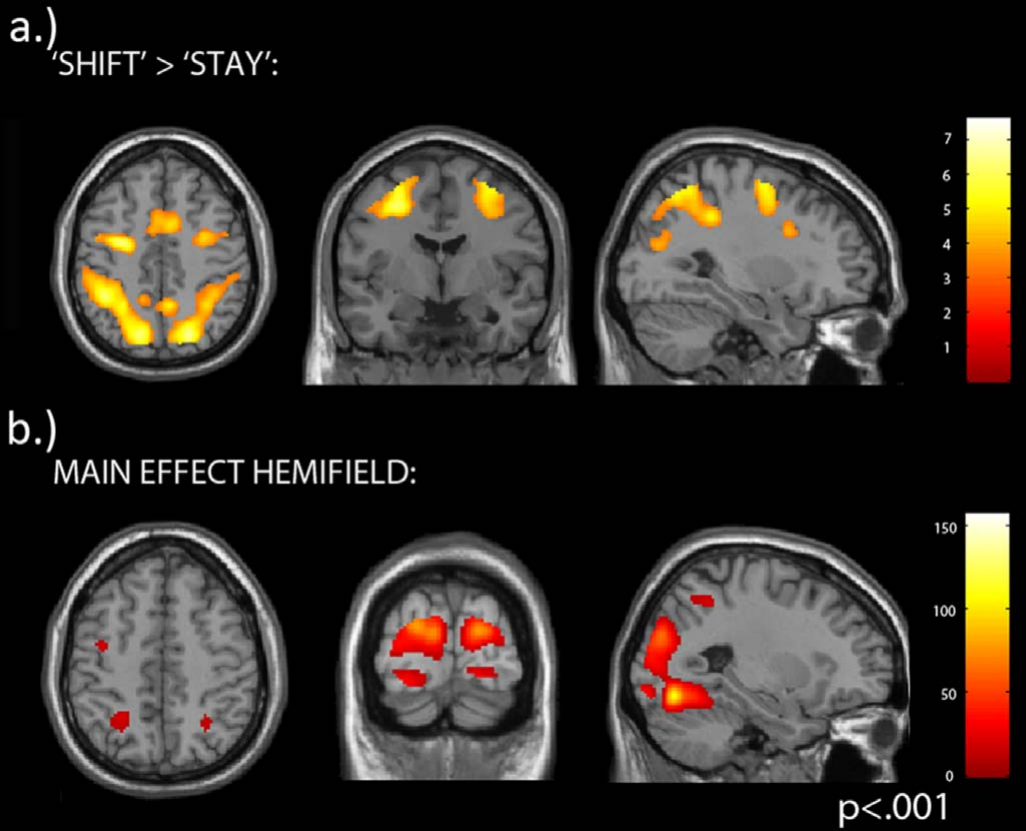
Activated clusters when contrasting ‘shift’ versus ‘stay’ mini-blocks **a)** and main effects of hemifield (F contrast) **b)**.

We first investigated task-related BOLD activity drawn from data acquired during the first pre-TBS/Sham blocks, which were not included in the subsequent analysis. At the whole brain level, a random-effects analysis with a contrast between shift-cued versus stay-cued mini-blocks revealed significant clusters within a dorsal attention network (bilateral FEF, IPS/IPL and SPL) and a more ventral attention network (right TPJ, right Insula/IFG and bilateral SMG). We also found activations in bilateral DLPFC clusters and bilateral Putamen (see Fig. 4a and Table 1; p<.001, uncorrected). For the reverse contrast (stay>shift) we found activated clusters in the precuneus and bilateral parahippocampal gyri. The task also activated contralateral visual cortex, extending to IPS and FEF, depending on the attended hemifield (see Fig. 4b and Table 2 for main effects of hemifield (F-contrast; p<.001, uncorrected)).

### 3.3 TBS effects on BOLD within attention networks

**Fig. 5:**
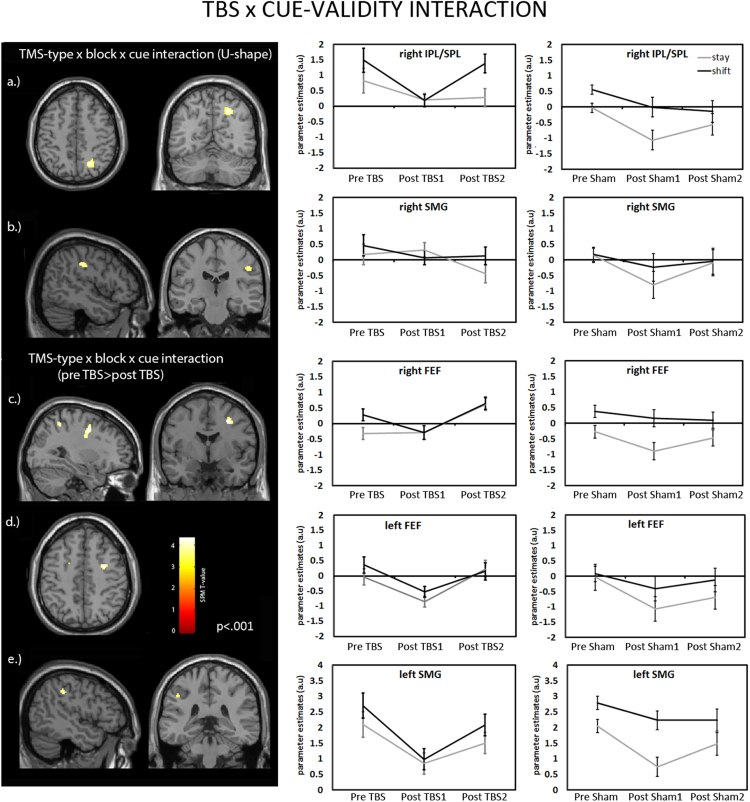
**a–b)** Displayed are regions which showed TBS effects on BOLD reflecting the behavioural U-shape pattern: a *decreased* activation following right FEF TBS as compared to Sham, specifically for ‘shift’- compared to ‘stay’ miniblocks, with recovery during the second block post TBS. Responsive clusters were found in right IPL (peak 30 −48 52) extending into right SPL (peak 24 −60 48) and right SMG (peak 48 −22 36). Right panels show extracted parameter estimates for the TBS and Sham data for illustration. **c–d)** Displayed are regions which showed TBS effects on BOLD that showed a prolonged impact (into the second block post TBS): a *decreased* activation following right FEF TBS as compared to Sham, specifically for ‘shift’- as compared to ‘stay’ miniblocks. Responsive clusters were observed in the right FEF (peak 28 −2 44), the left FEF (peak −16 6 50) and the left SMG (peak −48 −34 40). Right panels show extracted parameter estimates for the TBS and Sham data for illustration.

The main task effects (shift vs stay) thus confirmed that in addition to the VAN, also the FEF and other regions in the DAN are recruited following attention shifts. Based on the behavioural results, which demonstrated that TBS to right FEF temporarily impaired task performance particularly following a shift-cue as compared to trials following a stay-cue, we asked the question how the right FEF influences other regions within the recruited networks specifically on miniblocks following a shift-cue. Since we found a U-shaped behavioural pattern - disrupted performance on shift trials immediately following right FEF TBS, with a recovery during the second block post TBS - we applied a 3-way ANOVA to the fMRI data (TBS-type, blocks and cue-type) that reflected this behavioural pattern, to search for those brain regions that are initially suppressed by TBS during shift- compared to stay-miniblocks, but show recovery during the second block. More specifically, we tested for a 3-way quadratic interaction between cue-type and the blocks pre TBS and post-TBS1 with an opposite interaction during recovery, between the blocks post TBS1 and post -TBS2. This was compared against the reverse contrast applied to the Sham data (contrast: TMS-type (TBS – Sham)×block (2×post-TBS1 – (pre-TBS+post-TBS2))×cue-type (shift - stay)). Results are presented in Fig. 5 (masked for main effects of shift- versus stay). Regions of interest within the DAN, VAN and striatum (shift>stay contrast) were used to test for significance (see Methods and Supplementary Materials Fig. S4). Significant clusters responding to this interaction were found in right IPL extending to SPL and right SMG (see Fig. 5a,b; p<.05 small volume corrected). Extracted parameter estimates for the TBS and Sham conditions are shown for illustration (see right panels Fig. 5a,b).

To investigate whether activity was suppressed in additional regions, which did not completely recover but lingered across both blocks post stimulation, another 3-way ANOVA was applied to the fMRI data, which tested for any TMS-type x block x cue-type interaction effects post stimulation (TMS-type x blocks (2 x pre-TBS vs (post-TBS1+ post-TBS2) x cue-type), without taking the behavioural U-shape in account. This analysis revealed significant clusters in targeted right FEF, left SMG and to a lesser extent, left FEF (p<.05 small volume corrected; see Fig. 5c–e).

A final analysis including hemifield as an additional factor (3-way interaction: TMS-type x blocks x hemifield across both cueing conditions and for stay- and shift blocks separately) did not yield any significant clusters.

### 3.4 TBS effects on BOLD in visual cortex (ROI analysis)

**Fig. 6:**
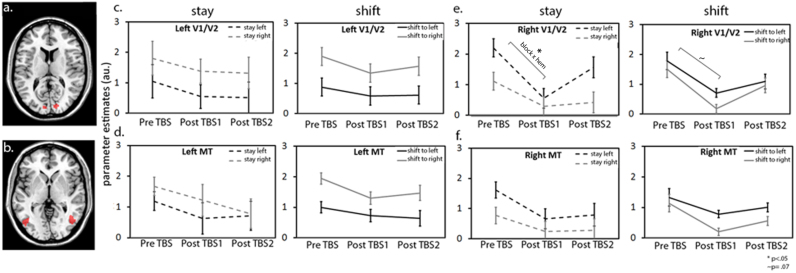
Extracted parameter estimates from task-activated ROI's in the visual cortex comprising V1/V2 (a.) and MT (b.) show impact of right FEF TBS only in the *right* visual cortex, particularly in V1/V2 (e.). Strikingly, a lateralised effect of TBS on neural responses was observed in V1/V2, when the focus of attention was maintained on the left hemifield target-stream (e. left panel). This lateralised impact was not observed for the ‘shift’ condition, where a general negative trend was detected across both attended hemifields (e. right panel). These effects recovered during the second session post TBS. Similar but non-signicant trends were present in the right MT (f.) and no clear effects were detected in the left hemisphere (c–d).

To investigate more subtle effects in the visual cortex, we identified regions of interest by contrasting all conditions against baseline combined with inclusive anatomical masks (including data from the first blocks pre TBS/Sham only, see Methods). This yielded clusters in V1/V2 and MT only (see Fig. 6a p<.001). For each participant, parameter estimates were extracted from these ROIs for each hemisphere for all miniblock conditions separately, and were subsequently averaged. To test for effects of FEF TBS, an ANOVA including the factors of TMS-type (TBS and Sham), blocks (pre TBS/Sham and post TBS/Sham1), cue-type and hemifield was applied on data for all ROIs separately.

#### 3.4.1 V1/V2

For V1/V2 in the right hemisphere, this analysis revealed a main effect of attended hemifield (F(1,13)=11,8 p=.005), reflecting stronger responses for contralateral as compared to ipsilateral attended targets and no other main effects. Although no interaction with TMS-type reached full significance (TMS-type×blocks×hemifield F(1,13)=2,4 p=.15), based on this trend we did run a subsequent analysis on the experimental data (FEF TBS) only. This analysis showed an interaction between block, cue and hemifield (F(1,13)=6,9 p=.02) indicating a differential effect of TBS depending on cueing condition and attended hemifield. Subsequent analysis separating data for each cueing condition, revealed an interaction between block and hemifield for stay-miniblocks (see Fig. 6; F(1,13)=5.9 p=.03) reflecting a (marginal) decrease in parameter estimates for left targets only (T(13)=−2.1 p=.06). For shift miniblocks a general negative trend was observed across both hemifields (see Fig. 6 F(1,13)=3,9 p=.07). These effects were no longer present during the second block post stimulation (block (pre vs post2)×hemifield×cue F(1,13)=.35 p>.5 ns.). This was reflected in a significant U-shape interaction when including all 3 blocks (blocks (2×post1 vs (pre+post2)×cue×hemifield (F(1,13)=11,6 p=.005)).

For the left hemisphere, the same ANOVA was applied (factors: TMS-type, block (pre, post1), cue and hemifield), which again revealed a main effect of attended hemifield (F(1,13)=16 p=.002) and no other main effects. No interactions with TMS-type reached full significance (TMS-type×blocks×hemfield F(1,13)=2,7 p=.12), but we again ran a subsequent analysis on the experimental data (FEF TBS) only. This revealed no main effect of block or any interaction with blocks, indicating no clear effects of FEF TBS on parameter estimates in the left V1/V2.

#### 3.4.2 MT

For the MT in the right hemisphere, the ANOVA (factors: TMS-type, blocks (pre, post1), cue and hemifield) revealed both a main effect of attended hemifield (F(1,13)=30 p<.0001) and of cue (F(1,13)=5,5 p=.037) and no other main effects. However, an interaction between TMS-type, blocks and cue (F(1,13)=7.7 p=.017) indicated an effect of TBS depending on cueing condition. A subsequent analysis on the experimental data (FEF TBS), revealed a marginal interaction between block, cueing condition and hemifield (F(1,13)=3,9 p=.07). Subsequent analysis separating data for each cueing condition, only revealed non-significant trends for decreased parameter estimates in stay-miniblocks (block F(1,13)=2.3 p=.15) and shift miniblocks (block F(1,13)=2.8 p=.12 block×hemifield F(1,13)=2.6 p=.13).

For MT in the left hemisphere, the ANOVA (factors: TMS-type, blocks (pre, post1), cue and hemifield) revealed a main effect of attended hemifield (F(1,13)=39,7 p<.0001) and no other main effects or interaction with TMS-type (p>.25).

In summary, only in the right hemisphere we observed effects of TBS over right FEF. Particularly in the right V1/V2, neural activity was reduced immediately following TBS, particularly for contralateral left- as compared to right hemifield attention following a ‘stay’ cue. These effects were no longer present during the second block post TBS and this U-shaped interaction pattern was not observed for the Sham data (p>.6) (see Supplementary Material Fig. S5). In the right MT, we observed a similar trend, but this did not reach significance.

### 3.5 TBS effects on functional connectivity

**Fig. 7:**
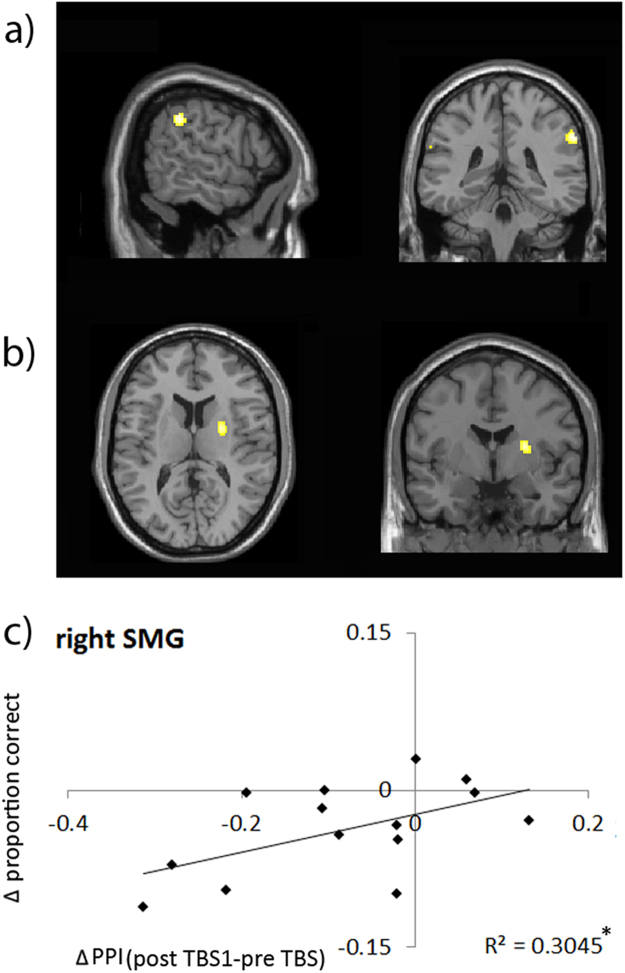
Displayed are clusters responding to the second level interaction contrast (U-shaped) between TMS-type, block and cueing type which was applied to the PPI estimates (Physiological time course: right FEF; Psychological Interaction: shift×stay) and revealed responsive clusters in a.) bilateral SMG and b.) right putamen. This indicates decreases in functional connectivity between the right FEF and these regions specifically during the shift condition as compared to the stay condition, which differed between TBS and Sham. c.) The TBS-induced decrease in PPI estimates in the right SMG (post<pre) correlated with a decrease in behavioural accuracy (proportion correct scores) specifically for the ‘shift’ condition.

To investigate how TBS to the right FEF influences functional connectivity with other regions in the attention networks and/or visual cortex, we performed a “psychophysiological interaction” (PPI) type of analysis (Friston et al., 1997). Time-series were taken from the targeted right FEF and tested for differences in covariation with any other brain regions when contrasting residual fluctuations in the shift- versus stay-conditions (i.e., after all main effects of task have been factored out). These PPIs were subsequently tested for interactions with TBS in a second-level analysis (see Methods). Fig. 7 displays voxel clusters that responded to the ‘U-shaped’ second-level contrast (again guided by the observed behavioural pattern): a *decrease* in PPI estimates (shift- versus stay-mini-blocks) immediately post TBS (post TBS1) compared to pre TBS, followed by an *increase* in the second session post TBS (post TBS2) versus the opposite pattern for Sham). Within the ventral attention network, this contrast revealed a significant cluster in the right SMG (p<.02 FWE small volume corrected). Furthermore, a significant cluster was found in the right putamen (p<.05 FWE small volume corrected). These results indicate that supressed neural activity in right FEF following TBS, temporarily disrupted functional connectivity specifically for shift-mini-blocks between right FEF and bilateral SMG and the right putamen, which recovered during the second block post TBS. No effects were observed within the visual cortex ROIs. A PPI analysis testing for decreased covariations with right FEF specific for stay-mini-blocks as compared to shift-mini-blocks did not yield any significant effects.

To test the impact of TBS-induced reduced connectivity, we correlated parameter estimates (from TBS data only), extracted from the responsive clusters with task performance. Specifically, we looked at correlations between TBS-induced differences in PPI parameter estimates (reflecting covariations greater for shift- compared to stay-mini-blocks) and TBS-induced differences on task accuracy (for shift- minus stay trials).

Contrasting post TBS1 with pre TBS, this analysis yielded a positive correlation between PPI estimates in right SMG and proportion correct scores (r=.55 p=.04). Note that we are looking at differences between shift- and stay-miniblocks. Hence, a positive correlation indicates that following TBS, a decrease in functional connectivity between right FEF and right SMG, specifically for shift-miniblocks, predicted the extent of impaired task performance following attention shifts.

No significant correlation with proportion correct-scores was found for extracted PPI estimates in the other clusters.

## 4 Discussion

### 4.1 FEF TBS impact on attention shifting

We demonstrated that TBS over right FEF impaired performance on an orientation discrimination task in both hemifields over a prolonged period of time (ie. for several subsequent targets) following attention shifts, which was not observed when attention was maintained. This TBS-induced deficit was temporary and recovered ca. 20 min post-stimulation. Our finding is compatible with a recent study reporting bilateral deficits in attention shifting (endogenously cued from fixation) when TBS was applied over the right FEF (Duecker et al., 2013). Other TMS studies (non-TBS) have also shown bilateral effects following disruption of right FEF (Grosbras and Paus, 2002, Grosbras and Paus, 2003, Ruff et al., 2006). Despite differences in paradigm, both our findings and these studies indicate that performance deficits following right FEF TMS are bilateral when disengagement from the current focus of attention (either current stream or fixation) and subsequent (re)orienting towards a new location is required.

FEF TBS did also have an impact on performance when participants were instructed to maintain the focus of attention, but only on the first target post-cue. Different from what we observed following an attention shift, a lateralised deficit in the left hemifield was observed immediately following a cue to maintain attention. Evidence for a selective lateralised impact following a stay-cue was strengthened by our fMRI findings in the visual cortex (see below). These findings combined are in concordance with the notion, as suggested by Duecker et al., that the FEF can behave according to the hemispatial theory during attention shifting (Heilman and Van Den Abell, 1980), with a bilateral influence of the right FEF (and as their study demonstrates, a more contralateral bias for left FEF). On the other hand, following a cue to maintain the focus of attention, the right FEF seems to employ a mechanism more akin to the ‘opponent processor’ or inter-hemispheric competition model (as described for the parietal cortex (Baylis et al., 1993; Hilgetag et al., 2001; Kinsbourne, 1977; Silvanto et al., 2009)). This effect was not observed for subsequent targets following the first target post stay-cue, and seems therefore to occur in interaction with the salient (alerting) cue.

It should be mentioned that the described effects on behaviour reflected the impact of TBS on inverse efficiency scores, chosen as a measure to account for speed-accuracy trade-off. Inspection of accuracy and response times separately revealed that these effects were mainly due to an impairment on accuracy, but an increase in response speed suggests that a speed-accuracy trade-off may indeed partly underlie the observed effects.

A surprising aspect of our results was the observation that the attention-shifting deficit was prolonged and was visible for at least four targets post-cue. This suggests a profound disruption of the underlying mechanism. Different sub-processes of attention shifting may have been affected by TBS, such as disengagement from the currently attended stream and/or (re)orienting to the contralateral stimulus-stream. TMS and EEG studies have shown that the FEF exerts a very early orienting role in target detection during visual search, indicating a leading involvement of the FEF in allocating behavioural salience (Blanke, et al., 1999) (O'Shea et al., 2004). A causal role for the FEF in facilitating disengagement from the current focus of attention was suggested in a TMS study, in which TMS to the FEF disrupted inhibition of return (Ro et al., 2003). Alternatively or in addition to this, the FEF may control both disengagement and orienting through attentional zooming, as TMS over FEF was shown to induce an inflexible focus of attention, impairing zooming-in or out from the current focus (Ronconi et al., 2014).

It could also be argued that TBS to the right FEF interfered with memory maintenance and/or processing of the cue itself, as it cannot be determined with certainty what part of the trial was most affected with offline TBS. Although this possibility cannot be excluded, we consider this unlikely because in that case shift- and stay trials would be expected to be similarly affected, which is not what was observed.

Below, we will discuss the different possibilities based on the observed effects of TBS to the right FEF on neural activity patterns.

### 4.2 Local and remote influences on neural responses of right FEF depending on attentional context

#### 4.2.1 Remote effects of FEF TBS following attention shifts in the ventral attention network and striatum

Specifically during shift mini-blocks, TBS to the right FEF suppressed neural activity in bilateral SMG and impaired functional connectivity between right FEF and SMG. We did not observe such effects in other regions of the ventral attention network. Previous TMS studies have shown that the SMG is critical for attention shifts between visual stimuli in different locations (Chambers et al., 2004, Rushworth et al., 2001). Moreover, inter-hemispheric functional connectivity between left- and right SMG is specifically correlated with a disengagement deficit in the acute phase of neglect (Lakatos et al., 2007). Our data indicate that neural processing within left- and right SMG is influenced by the right FEF and that functional connectivity between the right FEF and the right SMG drives attention shifts. Effects of TBS to the right FEF in both right- and left SMG, could explain the bilateral behavioural deficit we observe during attention shifts. Anatomically, signals may pass between the right FEF to the right SMG via the superior longitudinal fasciculus (SLF), which connects prefrontal cortex to both superior and inferior parietal lobes (Schmahmann and Pandya, 2007) and reach the left SMG via transcallosal pathways, perhaps through left FEF (Anderson et al., 2012) or subcortically.

Why do we not observe any effects in other regions of the ventral attention network that were identified when contrasting ‘shift’ versus ‘stay’ conditions, such as the right TPJ and IFG? The TPJ has been implicated in reorienting of attention by match/mismatch processing between expected and actual target appearance and the IFG mainly responds to a breach of expectation (Nobre et al., 1999, Vossel et al., 2006) or response inhibition (Chambers et al., 2007). The absence of effects of FEF TBS in the TPJ and/or IFG suggests that the right FEF does not modulate activity in these regions in a causal manner during spatial attention shifts in our task. This right lateralised part of the ventral attention network may only interact with the FEF during reorienting of attention after a *mismatch* between expected- and actual target-appearance has been detected (DiQuattro and Geng, 2011, Natale et al., 2010). In contrast, the cue-induced spatial attention shifts in our task involved disengagement from the current location, followed by orienting to the new location, but no mismatch operation was required as there were no invalid trials. The observed activation of the right TPJ and IFG could have served a separate mechanism in our task, such as task-set switching in response to the unpredictability of cue-appearance (Asplund et al., 2010, Lakatos et al., 2007).

We observed a disruption in functional connectivity between the right FEF and the right putamen. In an earlier study, which combined rTMS with fMRI, signals were perturbed in the putamen following rTMS to M1 during a non-spatial attention shifting paradigm (van Schouwenburg et al., 2012). The striatum is proposed to be involved in action selection by disinhibition of task-relevant motor programs and inhibition of competing ones (Mink, 1996). Similar (pre-motor) planning may be subserved by functional connectivity between the FEF and the putamen during spatial attention shifts in the current study.

#### 4.2.2 Local and remote effects of FEF TBS following attention shifts in the dorsal attention network

We show that attention shifts activate the dorsal attention network, which is in line with previous studies (Shulman et al., 2009, Yantis et al., 2002). Shift-specific TBS effects were found in the targeted right FEF, right IPL/SPL and to a lesser extent in the left FEF. All observed effects in these areas were *independent* of attended hemifield, suggesting that this signal may reflect a general ‘resetting’ operation (or circuit break), required to *disengage* from the current focus of attention, rather than orienting to the new location, which would be expected to depend on the location of the attended target. Several studies have demonstrated a pivotal role for the right SPL during attention shifting, which exhibits transient activation concurrent with shifts (Capotosto et al., 2013, Vandenberghe et al., 2001, Yantis et al., 2002). A slightly more lateral part of the right SPL, together with parts of the IPL, were activated in the current study, which may reflect different, more exogenous aspects of our paradigm as compared to earlier used tasks, due to high saliency of the colour cue and unpredictability of the cue occurrence.

#### 4.2.3 Remote effects of FEF TBS in the visual cortex

A lateralised effect of TBS was observed in the right visual cortex (V1/V2), where the neural response was suppressed by TBS when the focus of attention was maintained on the left hemifield target-stream. A non-lateralised impact was observed for the ‘shift’ condition. This finding corresponds with the lateralised behavioural deficit that was observed only for targets immediately following a ‘stay’ cue. Contralateral behavioural deficits are often associated with impaired functioning of the parietal lobe (IPS), as demonstrated with lesion- and TMS studies (Pascual-Leone et al., 1994). Our data indicate that a deficit in the processing of contra-lateral stimuli can also be caused by perturbation of neural activity in the right FEF. As the lateralised effect was only observed for the first target post stay-cue and not for subsequent targets in the stimulus stream, the effect seems to depend on the presence of a salient competing stimulus in the contralateral hemifield (colour cue). Future experiments could test whether this deficit is a true extinction-like effect.

## 5 Conclusion

Our results support a crucial role for the right FEF in mediating attention shifts, independent of attended hemifield. Importantly, we demonstrate that the right FEF drives attention shifts through functional connectivity with the SMG in the ventral attention network. We propose that this interaction may constitute a general (non-lateralised) signal to *disengage* from the current focus of attention. Furthermore, our findings indicate that the right FEF specifically controls selection of stimuli in the contralateral hemifield when attention is cued to be maintained, corresponding with lateralised modulation of stimulus processing in the visual cortex.

## Figures and Tables

**Table 1:** Shift vs Stay FEF (Frontal Eyefields); SEF (Supplementary Eyefields); PCC (Posterior Cingulate Cortex); SPL (Superior Parietal Lobe); IPS (Intra Parietal Sulcus); SMG (Supra Marginal Gyrus); IPL (Inferior Parietal Lobe); dlPFC (Dorso-lateral Prefrontal Cortex); TPJ (Tempero-Parietal Junction); MOC (Middle Occipital Cortex); SOC (Superior Occipital Cortex).

Cluster	Coordinates	T-score	P-value
Shift vs Stay:			
rSPL	20 -66 54	7.6	<.001
lSPL	-14 -64 56	7.4	<.001
lIPL	-40 -40 40	6.9	<.001
lFEF	-28 -6 48	6.3	<.001
rPCC	4 -56 54	6.2	
rIPL	36 -40 40	5.8	<.001
rDLPFC	50 6 34	5.8	<.001
lPCC	-10 -48 52	5.2	<.001
rFEF	32 -6 60	5.2	<.001
lMOC	-28–74 22	5.2	<.001
lIPS	-30 -50 46	5.0	<.001
lDLPFC	-46 4 26	5.0	<.001
SEF	4 8 50	4.8	<.001
rIPS	34 -50 44	4.75	<.001
rSMG	52 -32 40	4.4	<.001
lSMG	-58 -34 34	4.8	<.001
rMOC	34 -76 22	4.3	<.001
rTPJ	58 -36 26	3.9	<.001
rInsula	36 20 8	3.6	<.001
lPutamen	-22 8 -6	3.6	<.001
rPutamen	24 12 -2	3.3	<.001

Stay vs Shift:			
lPrecuneus	-8 -64− 23	4.27	<.001
lParahippocampal Gyrus	-22 -42 -7	3.51	<.001
rParahippocampal Gyrus	18 -18 -17	3.38	<.001

**Table 2:** Main effect hemifield (F-contrast).

Contrast	Cluster	Coordinates	Z-score	P-value
	L Fusiform Gyrus	-30 -74 -12	Inf	<.001
	R Fusiform Gyrus	30 -72 -10	Inf	<.001
	LSOC	-22 -84 28	Inf	<.001
	RSOC	44 -76 0	Inf	<.001
	L Calcarine Sulcus	-10 -92 18	Inf	<.001
	R Calcarine Sulcus	18 -88 20	Inf	<.001
	L V4	-24 -92 -12	5.74	<.001
	R V4	20 -88 -10	5.25	<.001
	lIPS	-26 -52 48	4.12	<.001
	lFEF	-38 -6 48	3.86	<.001
	rIPS	28 -56 48	3.59	<.001
