# Supplementary material for: Functional connectivity between prefrontal and parietal cortex drives visuo-spatial attention shifts
Source: Neuropsychologia. 2017 May;99:81–91. doi: 10.1016/j.neuropsychologia.2017.02.024 (PMC5415819; doi:10.1016/j.neuropsychologia.2017.02.024)
Supplement: Supplementary file 1 — Supplementary material [file mmc1.docx]

**Supplementary Material**

**
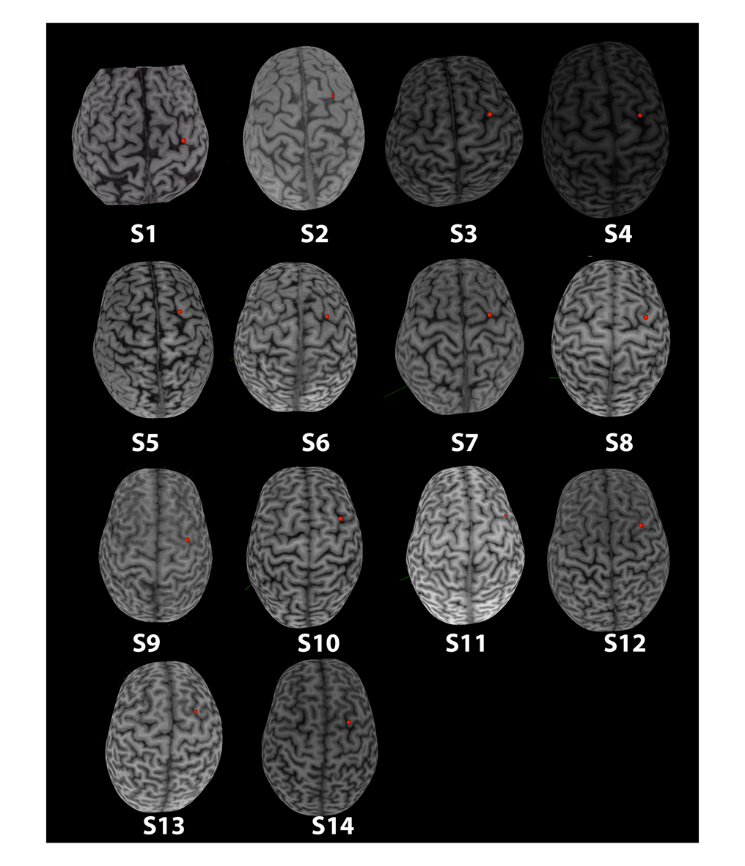
**

**Figure S1**

TBS sites per participant.


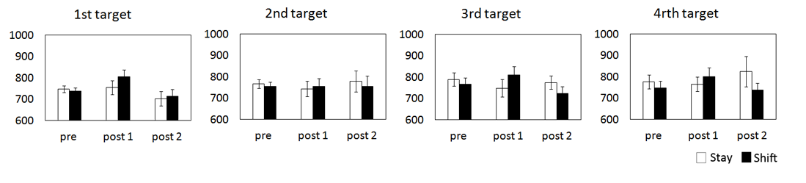


**Figure S2.** These graphs representing the impact of FEF TBS on the first four targets post cue separately, show a prolonged increase on the inverse efficiency score on shift trials immediately following TBS (post 1), still detectable for the fourth target post-cue.

**
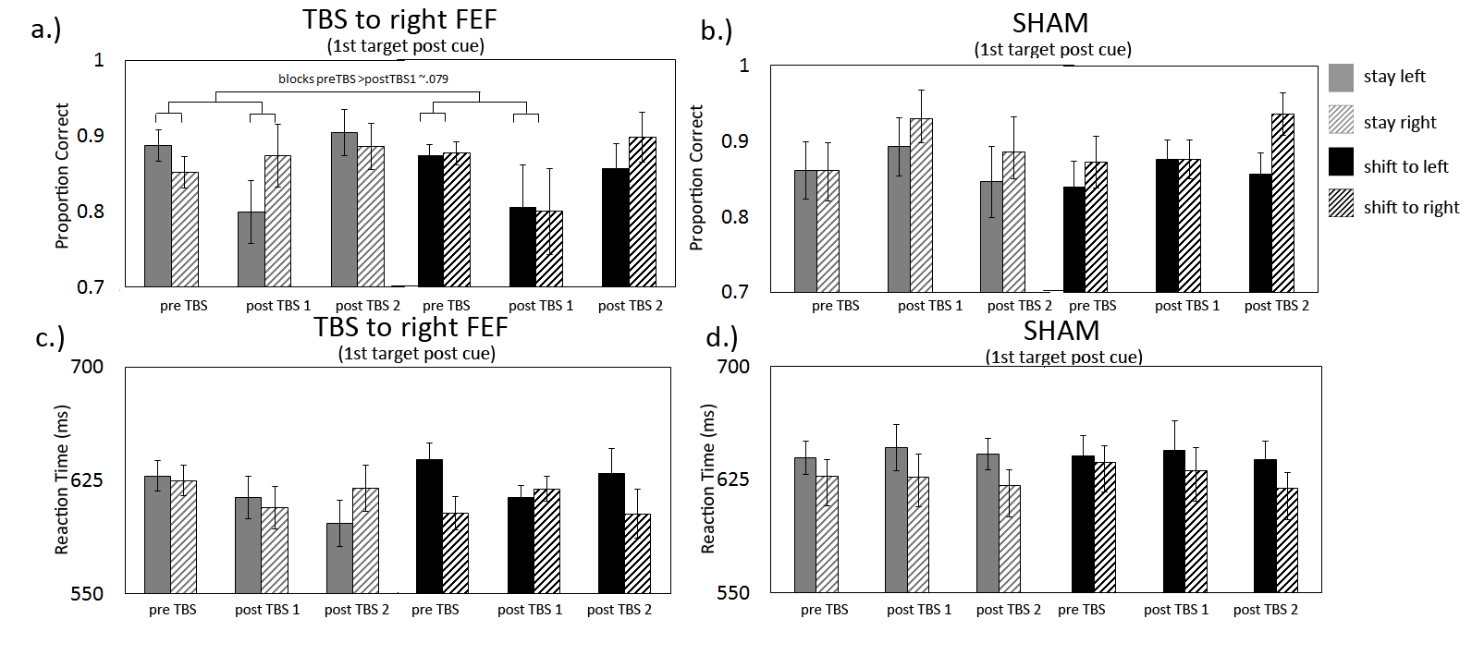
**

**Figure S3.**

The FEF TBS effects on inverse efficiency scores immediately following the cue (including data from the first target post-cue only) as reported in the main text were mainly due to an impact on proportion correct scores reflected in a marginal main effect of block. (a). No such effects were observed for the Sham condition (b). No significant effects of TBS were observed for reaction times.


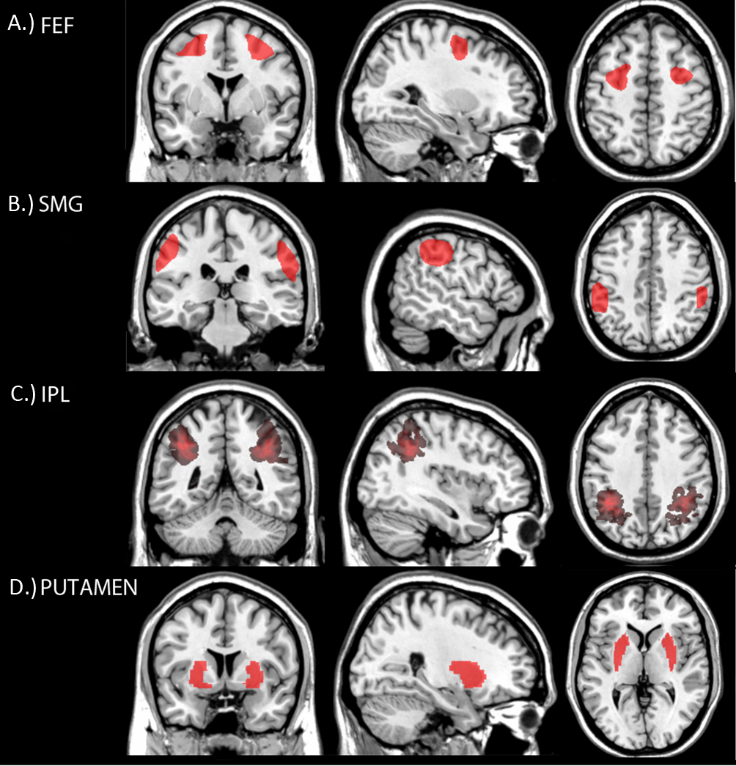


**Figure S4**

Masks used for small volume correction either derived from task activation (‘shift’ versus ‘stay’ contrast) for (a) FEF and (b) SMG or from probabilistic cytoarchitectonic maps derived from human post-mortem studies normalised into the SPM environment (Eickhoff et al.,2005) for (c) IPL and (d) Putamen.


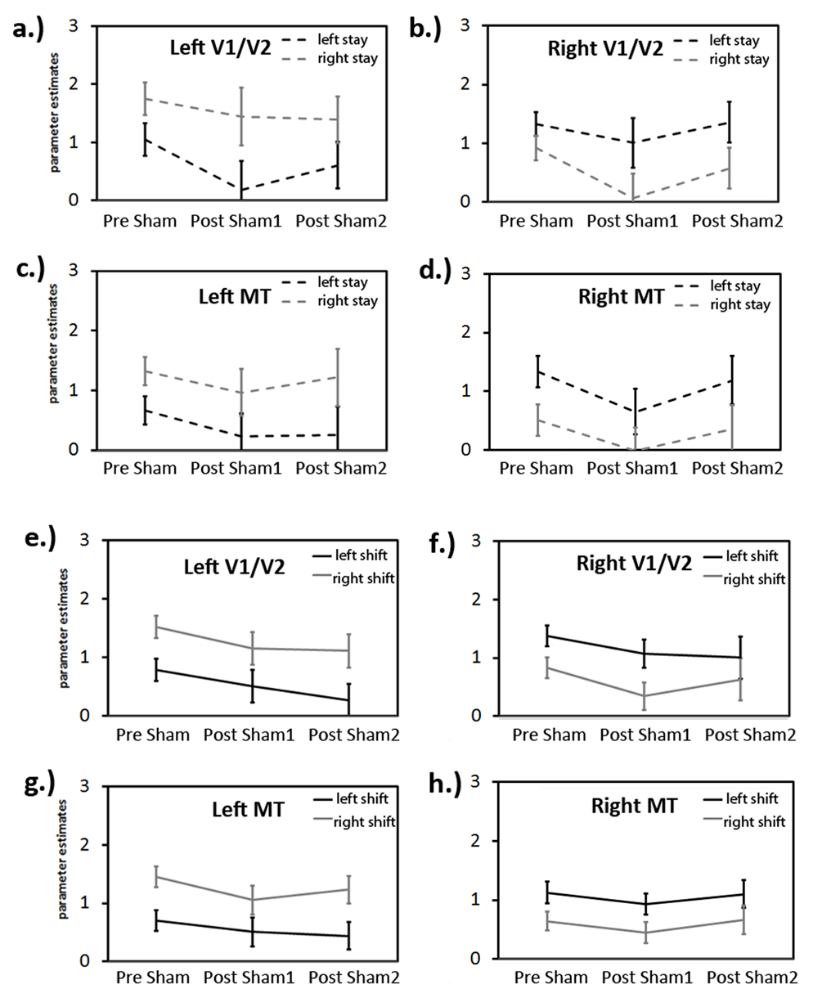


**Figure S5** No significant effects of Sham stimulation on neural responses were observed in the visual cortex ROIs.
